# Reasons why smartphone-alerted first responders abort missions: Findings from a sequential mixed-methods study

**DOI:** 10.1016/j.resplu.2026.101404

**Published:** 2026-07-03

**Authors:** Stephanie Stein, Carolin Braun, Bernd Reuschenbach, Stephan Prückner, Nadine Liebchen

**Affiliations:** a Department of Health and Care, University of Applied Sciences, Preysingstraße 95, 81667 Munich, Germany; b Institut für Notfallmedizin und Medizinmanagement (INM), LMU University Hospital, LMU Munich, Munich, Germany; c Arbeitskreis Notfallmedizin und Rettungswesen e.V. (ANR), Schillerstrasse 53, 80336 Munich, Germany; d Department of Anaesthesiology, LMU University Hospital, LMU Munich, Munich, Germany

**Keywords:** Smartphone-based first responder system, Smartphone-alerted first responder, Smartphone-based first responder alerting system, OHCA, Alert acceptance, Aborting missions

## Abstract

**Background:**

Smartphone-based first responder systems are implemented in 209 regions across Germany to reduce time to treatment in out-of-hospital cardiac arrests (OHCA). However, more than 20% of alerted responders abort a mission after accepting the alert. Understanding the underlying reasons is important for improving the prehospital chain of survival.

**Methods:**

We conducted a mixed-methods study comprising of (1) semi-structured interviews with smartphone-alerted first responders in Munich to identify and categorise reasons why missions are aborted, and (2) a subsequent quantitative online survey sent to all registered users of the *Mobile Retter* smartphone app in Munich to determine the frequency of these categories.

**Results:**

Four semi-structured telephone interviews identified six categories with two to three subcodes through qualitative content analysis, which informed questionnaire development. Based on this questionnaire, 334 responder feedbacks were analysed. Among responders, 130 (38.9%) reported aborting a mission after acceptance; 204 had never discontinued a mission. Lack of time advantage over dispatched emergency medical services was the most frequently reported reason for aborting missions (*n* = 89; 41.8%). An additional 21.6% reported excessive estimated distance, and 12.2% could not locate the emergency site. Further reasons included presumed medically unnecessary alarms and situational unavailability.

**Conclusion:**

Smartphone-alerted first responders were more likely to abort missions due to structural and informational factors rather than safety concerns. Optimising dispatch information, tracking and alert processes, improving emergency site localisation, and refining spatial alerting criteria may reduce discontinued missions and strengthen system effectiveness in urban areas.

## Graphical abstract

**Figure:**
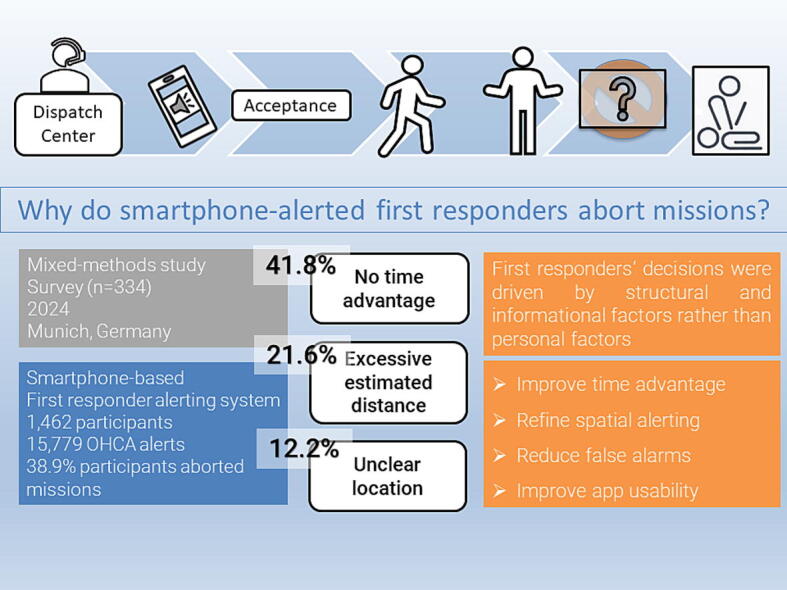

## Introduction

Smartphone-based first responder alerting systems are implemented in 209 regions^1^ across Germany to reduce time to treatment in out-of-hospital cardiac arrests (OHCA). Currently, five nationwide systems (“Corhelper”, “KATRETTER”, “Mobile Retter”, “Region der Lebensretter”) as well as regional alerting systems in Schleswig-Holstein (“Saving Life”), Saxony (“Ersthelfer Südwestsachsen”, using the Rescue Track app), and Bavaria (“Team Bayern Lebensretter”) are in operation. Their functionality follows a similar process: registered first responders are geolocated and alerted simultaneously with statutory emergency services within a regionally defined radius. Upon accepting an alert, they are navigated to the emergency location, and often also to the location of an AED, where they provide basic life support until emergency medical services (EMS) arrive.

In the Munich region, the “Mobile Retter” app (medgineering GmbH) has been in use since September 2021 as part of the cooperative project “München rettet Leben” (MrL). Alerts are triggered automatically based on predefined dispatch keywords, agreed upon by the project partners: “resuscitation”, “unconscious person”, “resuscitation child”, and “unconscious child”. Communication from the integrated dispatch center is unidirectional; additional information can be transmitted via a free-text field with limited character count. The classification of the Munich system, according to the standardised taxonomy for smartphone-based first responder systems proposed by Müller et al.,^2^ can be found in the additional files (Additional file A). As of September 2024, 1,462 medically qualified participants were active in the Munich region and 15,779 alerts were issued by the dispatch center. From April to September 2024, 332 missions with 385 participants (every alert is aimed at two participants) were accepted and 263 missions completed (acceptance rate: 11.3%; discontinuation rate: 21%; see Table 1).

**Table 1:** Mission statistics of “München rettet Leben” (MrL); source: project coordination team’s support portal.

	**Interviews**	**Survey**
Assessment time	10/23–04/24	04–09/24
Mission alerts (*N*)	2.319	2.939
Missions accepted (*N*; %)	245; 10.5	332; 11.3
Number of participants who accepted missions (*N*)	279	385
Participants who completed an accepted mission (*N*)	217	308
Completed missions (*N*; %)	184; 75	263; 79.2
Participants who discontinued an accepted mission (*N*)	49	74
Discontinued missions (*N*; %)	48; 19.6	69; 20.8

Similar discontinuation rates have been reported in other regions: KATRETTER Berlin 27%^3^; “Mobile Retter”- regions 2017–2018: 20%.^4^ Pommerenke et al. identified the most common reasons for aborting a mission as late arrival at the scene, accidental alert acceptance, navigational issues and safety concerns.^3^

The aim of this mixed-methods study was to qualitatively explore and subsequently quantify reasons why participants abort a mission within the Munich system in order to identify opportunities for system optimisation.

### Study design and methodology

A sequential mixed-methods design was applied, consisting of qualitative semi-structured interviews followed by a quantitative survey. Reporting for this observational study followed the STROBE guidelines (Additional File B). The study was approved by the Ethics Committee of Ludwig Maximilian University (reference no. 24-0238). Written informed consent was obtained from all interviewees. The study objectives and procedures were explained to all participants. Participation was voluntary, and consent could be withdrawn at any point during the interview. Collected data will be stored for ten years and secured against unauthorized access.

### Data collection

In April 2024 email invitations were sent to participants of the MrL project who had accepted yet aborted an alert the previous six months. Between late April and early June 2024, semi-structured interviews were conducted with participants who responded to the email invitation. The interviews explored situational conditions at the time of alert, first responder behavior during the mission, and reasons for aborting a mission. Mission discontinuation was defined as a first responder actively discontinuing a mission after accepting the smartphone alert. Subsequently, the project coordination team distributed a standardised survey invitation via email to all registered first responders between September 17th and October 14th 2024 (*N* = 1,462), asking them to complete a questionnaire.

### Qualitative analysis

**Fig. 1:**
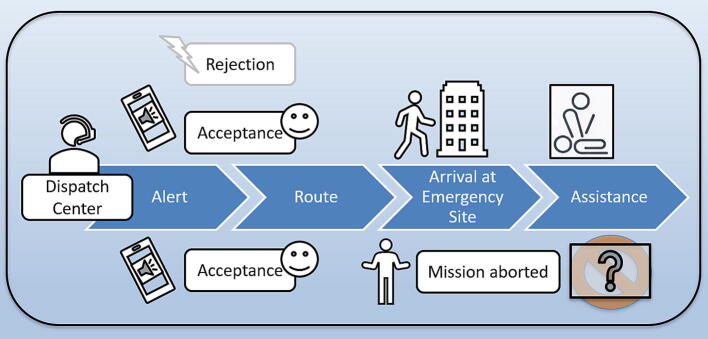
**Illustration of process steps**.

Data analysis was conducted using a deductive-inductive procedure based on Mayring’s approach.^5^ Four predefined process steps (Fig. 1) of a typical app-based response (alert, route, arrival at emergency site, assistance) served as main categories for the coding framework. Cited reasons for mission discontinuation were assigned to the corresponding process step and quantified by frequency. Intersubjective plausibility was verified through independent coding by a second coder. Transcription and analysis were conducted using MAXQDA (version 24.4.0).

### Structured questionnaire and quantitative analysis

The most frequently identified codes were used to develop a quantitative survey. An anonymous online questionnaire (SoSci Survey; Additional File C) was sent to all registered users in September 2024, with an 18-day deadline. Only fully completed questionnaires were included in the analysis. Descriptive analyses were conducted using Microsoft Excel (version 2410).

## Results

As of October 2023, there were just under 1,000 medically qualified participants in the Munich region. From October 2023 to April 2024 2,319 mission alerts were issued by the dispatch center (Table 1). 245 missions with 279 participants (every alert is aimed at two participants) were accepted and 217 missions completed (acceptance rate: 105%; discontinuation rate: 19.6%).

### Semi-structured interviews

In April 2024, invitation emails for participation in the qualitative interview study were sent to 37 first responders who had previously aborted at least one mission after alert acceptance. Four app users responded to the invitation and subsequently participated in the qualitative interviews. Gender distribution among the four telephone interviewees (10–20 min) was in balance. All interviewees had a medical background from either professional or volunteer experience. All had participated in multiple app-missions; completed as well as discontinued. Participants were first asked to compare their experience with the typical mission sequence shown in Fig. 1. All confirmed the depicted steps. Two interviewees added additional categories irrespective of the mission process: “app functionality” and “local EMS structure”.

The structuring content analysis followed Mayring's framework^5^ combining deductive and inductive category development. The deductive coding framework was derived from the literature and structured around the four steps as shown in Fig. 1 (Additional File D). During coding, relevant material emerged that fit the two additional categories noted by participants in the introductory question and were therefore integrated into the coding framework. The content analysis identified six categories with two to five codes each (Table 2).

**Table 2:** Results from semi-structured interviews.

**Category**	**Code**	***N***	**Citation**
Alert		**11**	
	Reason for alert not appropriate	6	*“…saw, that the reason for the alert was hypoglycemia …” (I2-10)*
	Self-protection	2	
	Not ready for deployment		
	– Professional obligations	2	*“In addition, I had an event at work at that time.” (I4-5)*
	– Night-time alert	1	

Route		**28**	
	Distance	10	*“…even 300 m. That kind of distance would take a very long time.” (I2-14)*
	EMS already en route	8	
	Preparation/not ready for deployment		
	– Time until departure	3	*“… has practically overtaken me on the way.” (I2-10)*
	– Mental preparation	5	
	Choice of means of transport	1	*“The threshold is already a certain…” (I2-24)*
	Night-time alert/transport issues	1	

Arrival at emergency site		**22**	
	EMS already on site	7	*“If the emergency service is already there, then I don’t get involved.” (I4-16)*
	Address/emergency site unclear	7	
	Rejection by relatives/bystanders	6	
	Not ready for deployment	2	*“You often dońt have gloves. Yoúre in a bit of an unprepared situation, in many respects.” (I2-26)*

Assistance		**23**	
	No assistance required	8	*“You can immediately tell it’s not an emergency. The person is responsive …” (I3-25)*
	Negative reaction from EMS	6	
	Self-doubt about capability	4	
	Unprepared/caught off guard	3	*“The emergency doctors… do look at you a bit sideways.” (I2-36)*
	Other people already provide help	2	

App Use		**8**	
	Usability		*“The app is not particularly precise.” (I2-40)*
	– Details only visible after acceptance	3	
	– Confusing information	3	
	User error	2	

Local EMS Situation		**8**	*“…because the EMS infrastructure is simply so well developed and fast.” (I2-47)*

Total		**100**	

### Quantitative survey results

The eight most frequent reasons for mission discontinuation were included in an online multiple-choice survey (Additional File C). A total of 334 fully completed questionnaires were analyzed (376 total responses; response rate 26%). See Table 3.

**Table 3:** Participants’ quantification of alerts and reasons for withdrawal.

**Number of questionnaires**	**334**	**Number of alerts**	***N***	**Participants have**	***N***	**%**

		None	48	Declined alerts	98	29.3
		1–3	185	Repeatedly declined alerts	73	21.9
		4–6	64	Discontinuation of missions	130	38.9
		7 or more	37	Never discontinued a mission	204	61.1

**Year of registration at MrL**	***N***	**Location during alert**	***N***	**Reasons for mission discontinuation**	N*	**%**

2021	56	City of Munich	207	EMS en route/already on scene	89	41.8
2022	73	Region of Munich	37	Distance	46	21.6
2023	108	Both	40	Address/emergency site unclear	26	12.2
2024	76	No response	50	Not ready for deployment	18	8.5
Unknown	20			No assistance necessary	15	7.0
No response	1			App use	10	4.7
				Reason for alert not appropriate	3	1.4
				Rejection/negative reaction	3	1.4
				Other	3	1.4

* *N* = 130 users gave 213 reasons.

Among responders, 98 had previously declined alerts, 73 declined repeatedly. A total of 130 (38.9%) reported discontinuing a mission after acceptance; 204 had never discontinued a mission (61.1%). See breakdown of reasons why participants abort missions in Table 3.

## Discussion

In this mixed-methods analysis, aborted missions by smartphone-alerted first responders in Munich were largely due to external circumstances rather than personal factors. Similar to prior studies, a high proportion (41.8%) arrived on scene at the same time or after the EMS. Additionally, 21.6% perceived the distance to the emergency location as too far to reach quickly and 12.2% were unable to locate the emergency site despite a provided address. A lack of operational readiness accounted for 8.5% of discontinuations. Medical misclassification contributed less frequently but remains a relevant target for process optimization. Safety concerns were rarely mentioned.

### Time advantage and distance to the emergency location

Reasons for aborted missions in Munich and Berlin are similar in type and frequency. The most frequent reason—“arriving at the same time or after EMS”—is also common in other regions (Berlin 59%; Freiburg/Breisgau region 32–56%; MR regions 52%).^3^, ^4^, ^6^, ^7^ At first glance, these findings may suggest that reducing the alert radius could improve the likelihood of first responders arriving before EMS.

To better understand this relationship, we can look at the results of our own simulation: While expanding the geolocation radius from 400 m to 600 m showed an increased alert acceptance rate (from 9% to 22%; March–June 2023), it simultaneously reduced the responders’ time advantage only 45% arrived before EMS. The app’s supposed walking distances (March 2023: 398 m; June 2023: 585 m) correspond with 01:23 min (March 2023) and 01:54 min (June 2023), yet actual first responder arrival times averaged 02:37 min (max. 07:24 min) in March and 3:33 min in June (max. 11:38 min). In contrast, the median EMS driving time in Munich was 06:45 min (IQR 04:50–09:05 min; Bavarian EMS Report 2022, p. 72, Fig. 44).

The discrepancy between estimated distance and substantially longer arrival times may have several reasons. The first is the app’s assumption of an average arrival speed of 10 km/h, derived from a mixed mode of transport (on foot/by bike/by car). The second suggests behavioral and environmental factors –mental preparation time, orientation, navigational obstacles– play a larger role than assumed. In densely populated regions with low current responder density (1.4/km2; target 10/km2), radius optimisation alone is insufficient.

These findings illustrate the structural challenge of balancing responder availability and response time. Improving the app’s tracking and alert processes as well as adjusting the alert radius (the second most common reason) might decrease the proportion arriving after EMS but would also reduce the pool of available responders. Expanding the radius increases responder availability but may reduce the likelihood of arriving before EMS, potentially contributing to frustration and future participation.

### Unclear emergency location

Locating the emergency scene is a frequent challenge in urban settings such as Munich and Berlin (Munich 12.2%; Berlin 17%^3^). Large housing complexes often require precise additional information. During the study period, supplementary information entered in the optional free-text field by dispatchers was not transmitted to app users because of a procedural issue within the dispatch system. However, app navigation itself remained primarily GPS-based, and the free-text field only served as an additional supportive information source. Communication between the dispatch center and the first responders is unidirectional, meaning that responders are unable to ask follow-up questions or clarify unclear location details. The absence of bidirectional communication constitutes a structural limitation of the app system. As long as responders cannot specify unclear locations, app users rely on manually entered additional information within the character-limited free-text field. A more consistent and structured use of this free-text field, as well as the implementation of bidirectional communication, could help reduce the responder cited 12.2% discontinuation rate owing to unclear locations.

### False alarms and mismatched dispatch codes

False alarms are mentioned in both qualitative and quantitative data. In 7% of cases, additional information after alert acceptance led responders to conclude that no intervention was required. Alerts in Munich are triggered by four dispatch keywords. More precise medical details might become available only after acceptance (free-text field), due to data protection constraints.^8^ If the dispatch center used a defined keyword catalogue in the free-text field suggesting that professional personnel are already on-site and/or the medical emergency does not include cardiac arrest, first responders wouldn‘t be alerted. The use of the free-text field is not mandatory for the dispatcher in Munich and there is no defined key word catalogue for the free-text field. Seven percent of aborted missions due to perceived unnecessary intervention indicate that the current filtering system of false alarms remains insufficient. The opportunity to get in touch with the dispatch center (bidirectional app) may help clearing those cases and thus decrease aborted missions.

The KATRETTER system Berlin uses six specified “cardiac arrest” codes and only two additional categories (“altered mental status following aspiration event”, “respiratory arrest after airway obstruction”) to avoid unnecessary alerts for unconscious individuals with preserved breathing. A more differentiated dispatch coding system could also reduce unnecessary first responder alerts and thus the number of aborted missions in Munich.

### Lack of operational readiness

Some missions were aborted due to personal circumstances (e.g., childcare, occupational responsibilities). Whether these represent accidental alert acceptance, curiosity, or other factors cannot be determined from the data. Pommerenke et al. reported 21% erroneous acceptances.^3^

The MrL system allows its users temporary suspension of responder availability. Munich’s fluctuating acceptance rate (April 2024: 10%, April 2025: 37%) may indicate that many registered users do not accurately reflect their true operational readiness. Availability settings—such as weekly readiness schedules—could help prevent inadvertent acceptances and improve alert efficiency.

### Role of the first responder

Interviewees occasionally described uncertainty regarding their role and negative reactions from bystanders or EMS personnel. However, previous research has shown that emergency physicians generally consider lay responder involvement beneficial.^9^ Public awareness campaigns, the use of the digital first responder identification (stating qualification) within the app, and clear communication regarding responsibilities may improve the interprofessional cooperation on scene.

There is substantial recruitment potential: 47% of registered MrL users do not complete the registration process. Reasons seem to be the complicated registration process as well as the fact that users may be lay persons and do not qualify for participation yet. All Munich partners support a planned expansion of the program to include lay responders. Yet this first requires improvements in the registration process and general app usability by the app provider.

### Limitations

This study has several limitations. First, it identifies factors contributing to mission discontinuation within a single urban smartphone-based responder system. Application to other settings—particularly rural regions—is limited due to differences in EMS structures. Second, voluntary participation may have introduced self-selection bias, as more dedicated first responders are likely to both accept more missions and participate more frequently in surveys. Responders who accept a higher number of alarms are also more likely to abort missions, which may partly explain why 38.9% of survey participants reported having aborted at least one mission, compared to 21% of accepted missions that were not completed according to system data. Third, the analysis relied on self-reported reasons for withdrawal, which may be affected by recall bias and social desirability. Fourth, qualitative interviews were conducted with a subset of responders who may not represent the majority of profiles, which could lead to a selection bias Fifth, this study has a small sample size of four interviews, which restricts the transferability of findings and does not allow for a fully conclusive claim of saturation. However, the homogeneity of the sample — all participants shared the same role and operational context — provides partial methodological justification for this approach.^10^ Future research should replicate these findings with larger and more diverse samples. Sixth, during the study period, supplementary information entered into the optional free-text field was not transmitted to app users because of a procedural issue within the dispatch system. Although app navigation remained primarily GPS-based, this may have influenced responders’ perception regarding the availability of mission-related information. Seventh, sociodemographic data were not collected due to the research focus on the qualitative analysis and subsequent quantification of aborted missions. This decision was based on methodological and data protection considerations, with the aim of ensuring participants’ anonymity and minimizing responder burden. Consequently, no conclusions can be drawn regarding sociodemographic differences.

### Implications for practice

The findings provide initial insights into priority areas for process optimization.

• A substantial proportion of missions do not render time advantage, indicating the need to systematically reassess the app’ s tracking- and alert processes, spatial alerting criteria, and navigation support.
• False alarms and medical misclassification should be monitored regularly in cooperation with dispatch centers to minimize unnecessary alerts and preserve responder motivation.
• Bidirectional communication, the consistent and structured use of the free-text field and availability settings that improve the actual readiness might increase mission effectiveness in Munich.

## Declaration of generative AI and AI-assisted technologies in the manuscript writing process

During the preparation of this work the authors used OpenAI in order to improve readability as non-native English speakers. After using this tool, the authors reviewed and edited the content as needed and take full responsibility for the content of the published article.

## CRediT authorship contribution statement

**Stephanie Stein:** Writing – review & editing, Writing – original draft, Visualization, Methodology, Investigation, Formal analysis, Data curation, Conceptualization. **Carolin Braun:** Writing – review & editing, Validation, Project administration, Funding acquisition, Formal analysis, Conceptualization. **Bernd Reuschenbach:** Writing – review & editing, Supervision, Methodology. **Stephan Prückner:** Writing – review & editing, Supervision. **Nadine Liebchen:** Writing – review & editing, Writing – original draft, Visualization, Validation, Project administration, Methodology, Investigation, Formal analysis.

## Funding

The City of Munich and the administrative district of Munich fund the project *München rettet Leben*.

## Declaration of competing interest

The authors declare the following financial interests/personal relationships which may be considered as potential competing interests: Nadine Liebchen reports financial support was provided by City of Munich, Department of Information Technology. Carolin Braun reports financial support was provided by City of Munich, Department of Information Technology. Nadine Liebchen reports a relationship with City of Munich, Department of Information Technology that includes: consulting or advisory. Carolin Braun reports a relationship with City of Munich, Department of Information Technology that includes: consulting or advisory. If there are other authors, they declare that they have no known competing financial interests or personal relationships that could have appeared to influence the work reported in this paper.

## Data Availability

The data sets used and analyzed in the current study are available from the corresponding author on reasonable request.

## Appendix A Supplementary material

Supplementary Data 1 File A: Reporting standard for describing first responder systems, smartphone alerting systems, and AED networks.

Supplementary Data 2 File B: STROBE Guidelines.

Supplementary Data 3 File C: Questionnaire.

Supplementary Data 4 File D: Coding categories and definitions.
