# Supplementary material for: Reasons why smartphone-alerted first responders abort missions: Findings from a sequential mixed-methods study
Source: Resusc Plus. 2026 Jul 3;30:101404. doi: 10.1016/j.resplu.2026.101404 (PMC13400656; doi:10.1016/j.resplu.2026.101404)
Supplement: Supplementary Data 1 — File A: Reporting standard for describing first responder systems, smartphone alerting systems, and AED networks. [file mmc1.docx]

## File A: Reporting standard for describing first responder systems, smartphone alerting systems, and AED networks

Table A1: Classification of first-responder system

| Items associated with the first responder system | | |
| --- | --- | --- |
| Category | Item |  |
| 1A Geospatial | Region | City and Region of Munich |
|  | Square km covered | 977 |
|  | Inhabitants | 1915000 |
|  | OHCA incidence | 65-126 (Fischer 2024) |
| 1B System Description | Name of the app | Mobile Retter |
|  | Organisation | Adesso AG |
|  | Relevant authorities | City of Munich/ Munich County |
|  | AED network | No |
|  | Number of FR (06/24) | 1323 |
| 1C Role of dispatch centre | Number of emergency dispatch centres | 1 |
|  | Systems in use | 1 |
|  | Activation | Through dispatch centre |
|  | Trigger mode | Automatically |
|  | Mission withdrawal | No contact options between dispatch centre and FR |
| 1D Maturity of responder system | Maturity | established |
| 1E Time | Daytime | 24 hours 365 days |
| 1F Role allocation | Number of FR alerted in a single mission | 2 |
|  | Roles in the system | 2 FR go to patient |
| 1G Activation criteria | Trigger for activation of the system | “Resuscitation Adult” “Adult unconscious” “Resuscitation Child” “Child unconscious”” |
|  | Exclusion criteria | Mesh term filter via infotext (limited number of characters) |
| 1H Delay time to the system activation | Delay time | No relevant delay |
| 1J Places alerted to | Sites | Public and private places |
|  | Sites FR are not dispatched to | Qualified personnel expected on scene, potential danger for FR |
| 1K Characteristics of FRs | FR groups | 2021-2022 EMS  2022- today: all medical professionals, police corps and trained corporate first aid* |
|  | On duty/off duty | FR’s responsibility in accordance with employer |
|  | FR duties | Provide first aid including BLS on the scene |
| 1L Additional features | Feedback from/report | Voluntary feedback protocol |
| 1M data reporting | How are data reported | System backend by the app owner, accessible for regional administrators |
| 1N Psychological protection of FR | Psychological support | Optional debriefing offer via local crisis intervention team |

*Emergency medical service personnel and emergency physicians; medical or dental healthcare professionals; physicians / dentists; volunteers in aid organizations/private emergency medical service companies or fire departments with appropriate training; police officers; paramedics, emergency first responders Alpha/Bravo of the German Armed Forces (Bundeswehr); mountain rescue services; water rescue personnel; fire departments; nurses and healthcare professionals; anesthesia technical assistants; pediatric nurses and healthcare professionals; midwives; geriatric nurses/elderly care nurses; medical students

Table A2: Classification of participants

| Items associated with the first responder network | | |
| --- | --- | --- |
| Category | Item |  |
| 2A Training | 1: Qualification | Any medical professional, including certified first responders* |
|  | 2: Revalidation | None |
| 2B Type of FR | 1: Inclusion/Exclusion | Age over 18 |
|  | Volunteer/Professional | Volunteers including professional responders in accordance with employer |
| 2C Availability | Location of FR | Not applicable |
| 2D Equipment | Equipment provided | None |

*Emergency medical service personnel and emergency physicians; medical or dental healthcare professionals; physicians / dentists; volunteers in aid organizations/private emergency medical service companies or fire departments with appropriate training; police officers; paramedics, emergency first responders Alpha/Bravo of the German Armed Forces (Bundeswehr); mountain rescue services; water rescue personnel; fire departments; nurses and healthcare professionals; anesthesia technical assistants; pediatric nurses and healthcare professionals; midwives; geriatric nurses/elderly care nurses; medical students

Table A3: Technical classification

| Items associated with the technology, algorithms and strategies | | |
| --- | --- | --- |
| Category | Item |  |
| 3A Technical availability | 1: Operating system | Android, iOS |
|  | 2: Critical alert | Yes |
| 3B: Location technology | 1: How are FR located | Mobile Phone Positioning System; GPS |
|  | 2: First responder tracking | Yes |
|  | 3: Position updates | Before an alarm |
|  | 4: Navigation | Map feature included |
|  | 5: AED visible in app | No |
| 3C: Algorithm | 1: Distance calculation | Distance by air  and travel distance |
|  | 2: Alerting radius | 120sec. travel distance to emergency in city; 300 sec rural region  Google Distance Resolver |
|  | 3: Mode of transportation | Average estimate 10 km/h |
|  | 4: Alarm procedure | 2 FR alerted, 2 FR go to the patient;  Search for FR is terminated after 240sec city/600 sec county |
|  | 5: Responding alarms | Accept/reject; Acceptance/withdrawal visible for backend team |
|  | 7: AED task | No |
| 3D: Legal | 1: Privacy restrictions | DSGVO/project T&Cs |
