# Supplementary material for: Reasons why smartphone-alerted first responders abort missions: Findings from a sequential mixed-methods study
Source: Resusc Plus. 2026 Jul 3;30:101404. doi: 10.1016/j.resplu.2026.101404 (PMC13400656; doi:10.1016/j.resplu.2026.101404)
Supplement: Supplementary Data 2 — File B: STROBE Guidelines. [file mmc2.docx]

## File B: STROBE Guidelines

| **Title and abstract** | 1a  1b | Sequential mixed-methods study  Structured summary provided |
| --- | --- | --- |
| **Introduction** |  |  |
| Background/rationale | 2 | Importance of smartphone alerted first responders  Improve outcome after OHCA |
| Objectives | 3 | Identify and quantify reasons for mission withdrawal by first responders after alarm acceptance |
| **Methods** |  |  |
| Study design | 4 | >Introduction |
| Setting | 5 | >Data collection |
| Participants | 6 | >Data collection |
| Variables | 7 | Mission withdrawal was defined as an active decision by the responder to discontinue the mission after alarm acceptance. |
| Data sources | 8 | >Data collection |
| Bias | 9 | >Limitation |
| Study size | 10 | Sample size was determined by feasibility rather than a priori power calculation |
| Quantitative variables | 11 | n.a. |
| Statistical methods | 12 | Descriptive statistics |
| **Results** |  |  |
| Participants | 13 | 4 interviews, 367 questionnaires received, 334 fully complete questionnaires analysed |
| Descriptive data | 14 | Table 3 |
| Outcome data | 15 | n.a. |
| Main results | 16 | >Semi-structures interviews  >Quantitative survey results |
| Other analysis | 17 | n.a. |
| **Discussion** |  |  |
| Key results | 18 | >Discussion |
| Limitations | 19 | >Limitations |
| Interpretation | 20 | >Discussion |
| Generalizability | 21 | >Limitations |
| Other |  |  |
| Funding | 22 | No direct funding was provided to the participating researchers. The City of Munich and Munich county funds the project *München rettet Leben;* Publication fees are covered by the City of Munich. |
