# Supplementary material for: Reasons why smartphone-alerted first responders abort missions: Findings from a sequential mixed-methods study
Source: Resusc Plus. 2026 Jul 3;30:101404. doi: 10.1016/j.resplu.2026.101404 (PMC13400656; doi:10.1016/j.resplu.2026.101404)
Supplement: Supplementary Data 3 — File C: Questionnaire. [file mmc3.docx]

## File C Questionnaire

**Dear first responders,**

We would like to welcome you to the project evaluation of *München Rettet Leben*.
The purpose of this survey is to systematically collect your personal experiences as volunteer first responders. Specifically, we aim to identify factors that may lead to a mission withdrawal after an alert has been received and accepted. Understanding these contributing factors will help us detect operational challenges and potential barriers, enabling evidence‑based process improvements.

This survey is directed at all first responders who have received at least one alert since joining the program.

**1. In which year did you join the *München Rettet Leben* program?**

Please select:

- 2021
- 2022
- 2023
- 2024
- Do not know

**2. How many alerts have you received since joining the program?**

- None
- 1–3
- 4–6
- 7 or more

**3. In which areas did the alerts occur?**

*If you are unable or unwilling to provide this information, please enter “0”.*

- City of Munich: ___*number of alerts*__________
- Munich District: ___*number of alerts*__________

**4. Have you ever declined an alert?**

- Yes, once
- Yes, several times
- No

**5. If you answered “Yes, several times” to question 4: Please indicate the number of alerts you declined.**

*If you are unable or unwilling to provide an estimate, please enter “0”.*

Number of declined alerts: ___________________

**6. Have you ever discontinued a mission after accepting an alert?**

- Yes, once
- Yes, several times
- No

**7. If you answered “Yes, several times” to question 6: Please indicate the number of missions you discontinued.**

*If you are unable or unwilling to provide an estimate, please enter “0”.*

Number of discontinued missions: ______________

**8. If you answered “Yes” to question 6: Please indicate the reasons for discontinuing the mission(s).**

- Emergency services were already en route or on scene
- Distance to the emergency location was too great
- Address or emergency location was unclear
- No medical assistance was required
- Alert reason/diagnosis did not warrant intervention
- Rejection or negative reaction from relatives, bystanders, or professional personnel
- Required preparation time (e.g., leaving the location, getting dressed, orienting oneself, mental preparation)
- Application-related factors (e.g., app usability, user errors, interface clarity)
- Other: _________________________

**We sincerely thank you for your valuable contribution to this evaluation.**
