# Supplementary material for: Reasons why smartphone-alerted first responders abort missions: Findings from a sequential mixed-methods study
Source: Resusc Plus. 2026 Jul 3;30:101404. doi: 10.1016/j.resplu.2026.101404 (PMC13400656; doi:10.1016/j.resplu.2026.101404)
Supplement: Supplementary Data 4 — File D: Coding categories and definitions. [file mmc4.docx]

## File D: Coding categories and definitions

| Category | Definition | Differentiation (illustrative examples) |
| --- | --- | --- |
| Alert | The first responder has received the alert, makes the decision to accept it, and confirms the acceptance of the assignment in the app. The first responder is in the pre-departure phase, prior to beginning the journey to the scene. |  |
| Route | The first responder leaves their current location and travels to the emergency site. | The responder notices the EMS while still en route and terminates the operation |
| Arrival at emergency site | The first responder reaches the emergency location as indicated by the address. This does not necessarily mean that the patient is found immediately as additional local orientation or searching a large area may be required. | The EMS has already arrived at the emergency scene before the responder, which is why the FR decides to abort the mission upon arrival. |
| Assistance | The patient is located. The first responder assesses the situation and decides on the necessity and type of assistance to be provided. The assistance ends when the emergency medical service (EMS) takes over. The category also includes situations in which the FR reaches the patient but determines that no medical assistance is required and aborts the mission. |  |
| App use | Factors related to the functionality, usability, or situational handling of the app that influence the course of a mission. |  |
| Local EMS Situation | The characteristics of local emergency care infrastructure that influence the course of a mission. | A hospital or EMS station nearby reduces the response time of the emergency vehicles. |

## 
